# The Physician in Perplexity

**Published:** 1898-08-20

**Authors:** 


### The Hospital

A Journal of

The Medical Sciences and Hospital Administration.

Vol. XXIV.—No. 621.] Edited by Sir Henry Burdett, K.C.B., and Solomon C. Smith, M.D., M.R.C.P. [Aug. 20, 1898.

### THE PHYSICIAN IN PERPLEXITY.

Does the modern physician ever get time and opportunity to think his own thoughts? If the answer be that he does not, that is the same as saying that he does not get time and opportunity to be a man. For it is a proposition which will bear the strictest investigation known to the most critical logic, that no man can be a man who does not systematically think his own thoughts, and equally systematically will his own life. It is now a holiday time, and, perhaps, a few half informal grumblings and suggestions may be more in keeping with the medical mood of the moment than a formal article on a subject portentously important, but which may also be portentously dull.

The present writer has a grievance, a real, determined, angry grievance, against England, Germany, and America. These are the three countries which deluge medicine with physiology, good, bad, and indifferent, but mostly bad; which flood it with literature in the shape of medical books, with no soul of either science or practice in them, and which "evolute" new remedies, not by the score, but by the thousand annually, not one of which in fifty is worth even so much as a second thought. The inevitable effect of all this upon the average minds in the profession is, either to suffocate and so to paralyse them with what appears to be new knowledge, or else to so disgust the practitioner that he makes up his mind never to read at all, and on no earthly consideration whatever to experiment with a new drug. Medicine, in short, is swamped, drowned, stifled, and paralysed by innumerable exploiters within and without its ranks; exploiters whose only object is the shortest possible cut, not to fame and fortune, but to notoriety and pelf. Now, all this has an exaggerated sound about it. But indeed and indeed, however exaggeratedly it sounds, it does not express one-tenth part of the miserable truth. The steady practitioner, whose aim is to supply his patients with the very best resources which the science of the times can afford, finds that about half his busy hours are spent in the brain-wearing, and what should be quite unnecessary operation of separating the precious from the vile. And the vile is so very vile, and so overwhelmingly preponderant, that he almost wishes himself in the nether world, and permanently joined to the ranks of Sisyphus and Tantalus.

Well, what way of escape is there from all this; how may the conscientious physician get his head above water; how may the busy general practitioner stand up and feel himself a man? If it were permitted in a grave publication, and perhaps it might be in this present holiday time, the writer would give expression to some unparliamentary language here. For it is as obvious to him as noonday that there is no way of escape at all; at any rate, not for any practitioner who is not as strong and resolute in his head as Samson was in his body. And this is the reason: the profession is swamped with pedants; with persons in the consulting and special ranks who have a little money, no practice, and unlimited leisure; and these persons find their only consolation, the only salve of their disappointed self-love, in writing and reading all the rubbish which is annually poured out upon the profession, and so in persuading themselves that they are more learned and scientific than their better employed rivals. If it were not for the two or three thousand intolerable pedants in our ranks medical life would be worth living. As it is—well, a wise philosophy makes the best it can of the inevitable.

For the strong, the mentally strong and resolute, there is, however, a remedy, even for so all-powerful a plague as the epidemic of medical books and new remedies. The strong have learnt, what all diligent students learn in time, the art of selection. They do not, and they will not, read the books and the papers of the exploiter, the pedant, and the notoriety seeker. The study of a single page is generally quite sufficient to show what a book is made of. If it be pedantically expressed, if it be charged with a great show of learning, if it evinces a manifest anxiety to give the opinions of every other person, living or dead, who has ever written upon the same subject, then it is evident that it is a manufactured book. It is a pretty safe canon of literary criticism, especially of the medical order, that the book which gives publicity to everybody's opinion has no opinion of its own worth publishing. How much of other people's judgment did Lord Lister express when he was working out the antiseptic system of surgery and medicine? It was a frequent boast of the late Sir Andrew Clark, almost up to the time of his death, that he had "never written a book." What we need, almost more than anything else at the present moment in the medical profession, are two things: First, courageous independence of mind and judgment; and, secondly, a competent capacity for selection. Without these our practice has no rules, no certainty; it varies from day to day, and even from hour to hour; it is everywhere and it is nowhere. With them we shall daily place at the disposal of all our patients, if not the last new thing in drugs or the latest opinion in bacteriology, at least the best of the proved resources which the science of the time affords.

